# Cross-modal perception of human emotion in domestic horses (*Equus caballus*)

**DOI:** 10.1038/s41598-018-26892-6

**Published:** 2018-06-21

**Authors:** Kosuke Nakamura, Ayaka Takimoto-Inose, Toshikazu Hasegawa

**Affiliations:** 1 Department of Cognitive and Behavioral Sciences, Graduate School of Arts and Sciences, The University of Tokyo, Tokyo, Japan; 2 Department of Behavioral Science, Graduate School of Letters, Hokkaido University, Sapporo, Japan; 3 Center for Experimental Research in Social Sciences, Hokkaido University, Sapporo, Japan

## Abstract

Humans have domesticated many kinds of animals in their history. Dogs and horses have particularly close relationships with humans as cooperative partners. However, fewer scientific studies have been conducted on cognition in horses compared to dogs. Studies have shown that horses cross-modally distinguish human facial expressions and recognize familiar people, which suggests that they also cross-modally distinguish human emotions. In the present study, we used the expectancy violation method to investigate whether horses cross-modally perceive human emotions. Horses were shown a picture of a human facial expression on a screen, and they then heard a human voice from the speaker before the screen. The emotional values of the visual and auditory stimuli were the same in the congruent condition and different in the incongruent condition. Horses looked at the speaker significantly longer in the incongruent condition than in the congruent condition when they heard their caretaker’s voices but not when they heard the stranger voice. In addition, they responded significantly more quickly to the voice in the incongruent condition than in the congruent one. To the best of our knowledge, this is the first study to show that horses cross-modally recognized the emotional states of their caretakers and strangers.

## Introduction

Many social animals are thought to utilize emotional cues from conspecifics to effectively retrieve social and environmental information that helps maintain their social groups^1^. Several studies have investigated emotional perception in social animals. For example, chimpanzees (*Pan troglodytes*)^2^, crested macaques (*Macaca nigra*)^3^, and rhesus macaques (*Macaca mulatta*)^4^ perceive the facial expressions of conspecifics. Bonobos (*P. paniscus*) attend more to the emotional scenes of conspecifics than they do neutral scenes^5^, and tree shrews (*Tupaia belangeri*) recognize affect intensity in voices^6^. Moreover, horses (*Equus caballus*) respond differently to negative (separation) and positive (reunion) whinnies when they are produced by familiar individuals^7^. In addition, horses discriminate the facial expressions of their conspecifics and then show appropriate behavioral and physiological responses^8^. These findings suggest that horses exchange emotional information with each other and use it effectively.

Humans have domesticated social animals, such as dogs (*Canis familiaris*) and horses, which are considered companion animals as they have served as family or partners to humans for a long time. Dogs and horses have worked with humans for over 10,000 years^9^ and about 5,500 years^10^, respectively. Such companion animals have built a close and cooperative relationship with humans, which suggests that social signals, such as emotional information, play important roles in how these animals live and interact with humans. Therefore, how these animals perceive emotional signals and what roles the signals play are important questions to investigate. These emotions improve the relationships between humans and companion animals, protect the animals by inducing actions that are appropriate for the emotion, and improve animal welfare^11^.

Recently, studies of dogs’ perceptions of human emotions^12^ have shown that dogs distinguish a human’s smiling face from a neutral face^13^. Dogs investigated a strange box more actively when their owner’s face was smiling than when his/her face showed fear or neutrality, which suggested that dogs understand the meanings of human facial expressions^14^. However, these studies used the object choice task, which involved a training phase before the test phase and the use of similar stimuli. Therefore, they cannot exclude the possibility that the participant animals only showed stimulus control and that the behavior was due to conditioning. In contrast, training is not needed for the test employed in studies of cross-modal recognition using the expectancy violation method. Albuquerque *et al*. suggested that dogs cross-modally perceived human emotion using facial expression and voice^15^. In that study, the dogs were simultaneously shown a visual stimulus (human facial expression) and auditory stimulus (human voice). The dogs looked at the visual stimulus longer when its emotional value was consistent with that of the auditory stimulus than when the two values were not consistent. These results suggested that dogs cross-modally perceive human emotion by integrating visual and auditory signals, which they use in human-dog interactions.

Few studies have investigated horses’ perceptions of human emotions, even though horses are also companion animals. Horses are able to discriminate positive human faces from negative faces, and time-to-maximum heart rates is faster when they look at negative faces^16^. These results are consistent with those when conspecifics’ facial expressions are presented^8^, thus suggesting that the same mechanisms are involved in horses’ interactions with humans and horse conspecifics. Moreover, horses have rich facial expressions^17^, which implies that horses exchange emotional information through facial expressions with not only their conspecifics but also humans, discriminate human facial expressions, and understand their meaning. In contrast, auditory stimuli, as well as visual stimuli, play important roles in general interactions between horse conspecifics^18^. Horses have characteristic contact calls, or whinnies, and they use different whinnies for negative situations, such as social separation from other group members, and positive situations, such as social reunions with group members^19,20^. These findings suggest that horses exchange emotional information vocally.

In addition, horses integrate visual and auditory information to recognize individual conspecifics and humans^21–23^. In one study, horses were presented a visual stimulus of a familiar horse and then an auditory stimulus of the same horse or a different familiar conspecific. The horses looked toward the audio source significantly faster and for a longer time when the visual and auditory stimuli were not consistent compared to when they were consistent^21^. In the second study, horses were presented with two people and then their voices from the speaker which was at the center of the two people. When presented with familiar people, they could match the voice with the person^22^. These results suggest that horses cross-modally recognize conspecifics by integrating visual and auditory information^21^ and that cross-modal recognition is generalized toward conspecifics and familiar humans^22,23^. Therefore, horses are thought to use visual and auditory cues to recognize and communicate with humans. However, whether horses cross-modally perceive human emotions by integrating facial expression and voices is unclear.

In the present study, we used the expectancy violation method to examine whether horses cross-modally perceive human emotion. The expectancy violation method was developed in studies of infant cognitive development. Participants are presented with stimulus A1 and then either stimulus A2, which is expected from stimulus A1, or stimulus B2, which is not expected from stimulus A1. If the subjects expect stimulus A2 from stimulus A1, an expectancy violation occurs when the subject is presented stimulus B2. Generally, stimulus A1 is visual and stimulus A2 and B2 are auditory in studies of cross-modal recognition. If stimulus A1 is a smiling face, stimulus A2 is expected to be a voice with a positive emotion and stimulus B2 should be a voice with negative emotion. Thus, the expectancy violation occurs when the participants hear stimulus B2 after looking at stimulus A1. When the participants hear stimulus A2 after looking at stimulus A1, the expectancy violation does not occur. The participants’ responses toward stimulus B2 are expected to differ from those toward stimulus A2. Therefore, this method is useful because it excludes the ambiguity associated with the presentation of perceptional information of a single modality as distinguishing between individual recognition and more general discriminative abilities is difficult. Moreover, it allows for naturalistic studies of environmental flooding in response to various perceptional stimuli^24^.

In the present study, we investigated whether horses cross-modally perceived human emotion using the expectancy violation method. Moreover, we tested whether the familiarity between the participant horse and human facilitated perception. The behavioral and physiological [heart rate (HR)]^25^ responses of each horse were measured while they were shown a human facial expression and then heard a human voice with either a positive or negative emotional value. Horses are thought to respond appropriately to negative emotions of high arousal by increasing their arousal^16^ (see also Briefer *et al*.^19^). If horses cross-modally perceive human emotion, the horses were expected to look for a longer period, respond faster to the speaker, and have a higher HR when they were exposed to incongruent stimuli compared to congruent stimuli. The expectancy violation effect was expected to be bigger when stimuli from their caretaker were presented if familiarity facilitated the horses’ cross-modal perception of human emotion because horses discriminate familiar people from strangers and match the face of a familiar person with his/her voice^21^. Additionally, if horses learn to interpret human facial expressions during their interactions with humans and, especially, predict negative events by visualizing negative human facial expressions, they were expected to respond faster and look toward the speaker longer when the visual stimulus was negative than when it was positive.

## Materials and Methods

Nineteen horses (*E. caballus*) [mean ± standard deviation (SD) age, 14 ± 6.2 years; 18 geldings and 1 mare] were recruited from the equestrian teams of the University of Tokyo and Tokyo University of Agriculture and Technology (Tokyo, Japan) between August 2016 and December 2016. All horses knew their caretaker for at least three months and interacted with him/her daily.

Stimuli were collected from the caretakers and strangers who were unfamiliar with the participant horses and the same sex as the caretaker. The caretakers were members of the equestrian team who rode and cared for their horses. Photos of positive (happy) and negative (angry) facial expressions were shown on a screen in front of the horses. The size of the stimulus faces on the screen was A3 (42 × 29.7 cm). The facial expressions were validated using Facial Action Coding System descriptions^26^. Voice stimulus recordings of the nickname of the participant horse were played after the photos of the facial expressions were shown, and they were positive (gentle) or negative (scolding) voices. The auditory stimuli were recorded in the rooms next to each stable. The humans were instructed to imagine they were praising the horse while recording positive stimuli or scolding the misbehaving horse while recording negative stimuli. The mean ± SD loudness of the stimulus voices from the participant horse’s location was 67 ± 2.1 dB for the gentle caretaker voice, 67 ± 2.1 dB for the scolding caretaker voice, 67 ± 2.4 dB for the gentle stranger voice, and 68 ± 1.9 dB for the scolding stranger voices of the strangers. The mean ± SD length of the stimulus voices was 5.4 ± 0.67 s. Their length differed according to the lengths of the horses’ nicknames, which ranged from 4–8 s (see the Supplementary Electronic Information).

**Figure 1:**
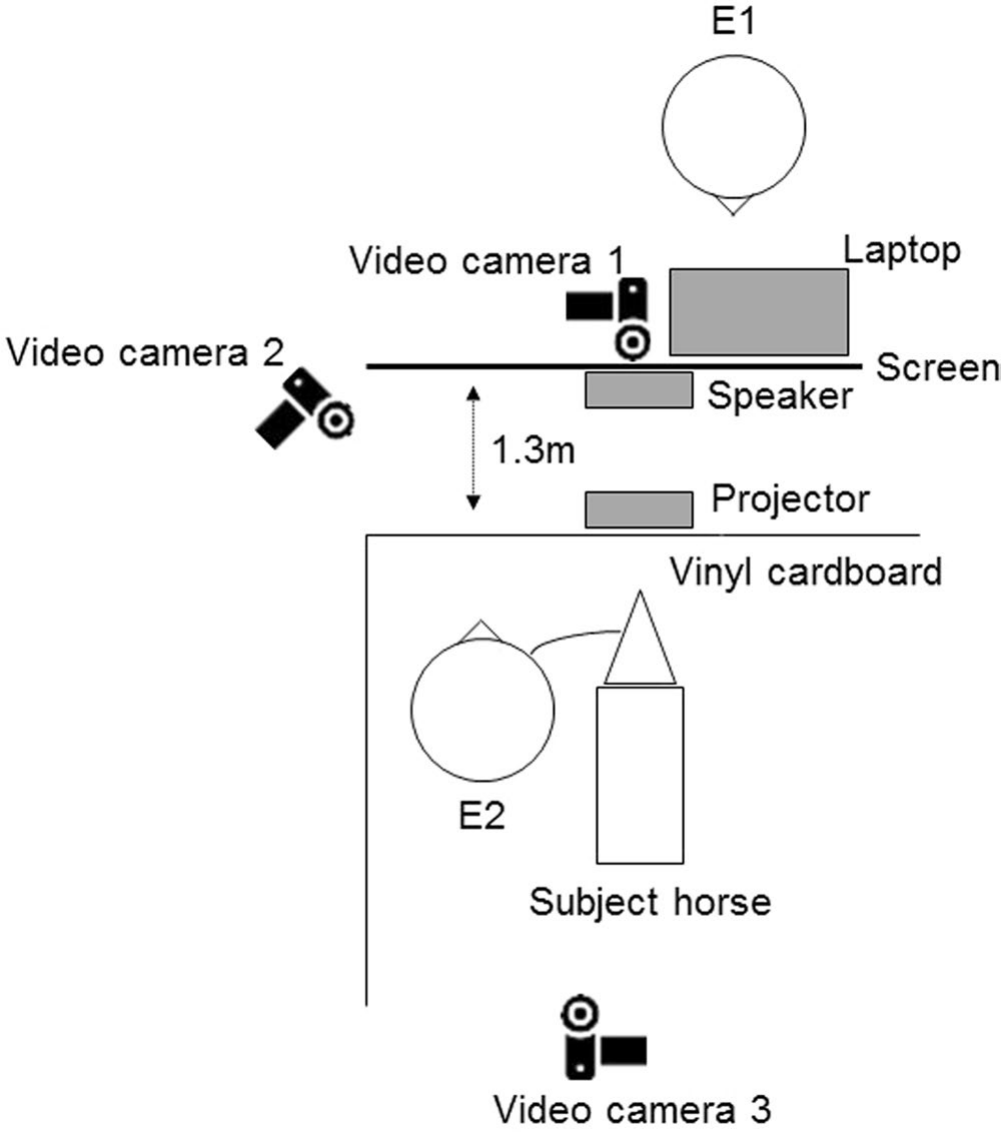
The experimental set-up. Visual stimuli were presented on the screen from the projector. Auditory stimuli were presented from the speaker.

The experiments were conducted either in the stable block at the University of Tokyo or Tokyo University of Agriculture and Technology. The experimental spaces had similar set-ups (Fig. 1). The screen on which the visual stimuli were presented was 1.3 m outside the experimental space. The projector, which was located just outside the experimental space, was hidden from the horses with cardboard. The subject horses were habituated to the space until their HRs stabilized and they stopped freezing and/or snorting in fear.

Our procedures were based on previous studies of the cross-modal recognition of human individuals by horses^23^. The trials were conducted in the stables by two experimenters. Experimenter 1 (E1) was hidden from the subject horses behind the screen, and he/she manipulated the laptop (SVD13238EJB VAIO; Sony Corporation, Tokyo, Japan) and controlled the presentations of the stimuli. Experimenter 2 (E2) took the participant horse to the experimental space and stood by it during the trial. E1 and E2 were not always the same person. Seven people acted as E1, and eight people acted as E2. They were equestrian team members. In the test trials, E2 first took the participant horse to the experimental space, turned its face toward the screen, and waited until its HR returned to rest levels (32–45 bpm^27^) or no longer changed over 10 s. The test trial then started when E2 began to record the HR. At the same time, E1 operated the laptop to present the stimuli and started the program. The program consisted of 15 s of a blank screen, 30 s of the visual stimulus presentation, 15 s of the blank screen again, and then the auditory stimulus presentation. E2 kept the lead loose and looked down after starting the presentation of the visual stimulus. The test trial ended 15 s after the presentation of the auditory stimulus, and E2 then left the experimental space with the participant horse. During the trial, E2 stood by the participant horse and manipulated the lead to face the horse towards the screen until the presentation of the visual stimulus. Each trial was recorded with three video cameras (two HDC-TM25, Panasonic Corporation, Kadoma, Japan; and one HDR-XR350; Sony Corporation) from the front, back, and left front, and HR was measured with a monitor (V800; Polar Electro Oy, Kempele, Finland).

We used a 2 (emotional congruency) × 2 (familiarity) × 2 (emotional value of the visual stimulus) within-subject design in this experiment. The independent variables were emotional congruency (congruent or incongruent), familiarity (caretaker or stranger), and emotional value of the visual stimulus (positive or negative). The dependent variables were response latency, total looking time, and HR difference between immediately before and 15 s immediately after presentation of the auditory stimulus. The horses participated in one trial per day and one trial per condition, which totaled eight trials per horse. The order of the trials was counterbalanced across the horses. The intertrial intervals were at least 2 d to prevent habituation. Eleven trials were excluded from the analyses because the participant horses did not look at the visual stimuli or the stimuli were not correctly displayed due to technical issues.

The horses’ behavioral responses (total looking time and response latency) were coded from each frame (1/30 s) of the video using PowerDirector 14 software (64 bit; CyberLink Corporation, New Taipei City, Taiwan). Total looking time was defined as the total number of frames in which the subject horses were looking toward the screen in front of the speaker from the moment that the auditory stimulus was presented to the end of the trial. Response latency was defined as the number of frames until the subject horses paid attention to the screen after the auditory stimulus was presented (see the Supplementary Electronic Information). The analyses of 27 of the 152 videotapes by a second rater resulted in interobserver reliabilities of 0.943 (Pearson’s *r*, *p* < 0.01) and 0.929 (Pearson’s *r*, *p* < 0.01) for total looking time and response latency, respectively.

A linear mixed model was used to examine the effects of emotional congruency, familiarity, and visual stimulus emotional value. Data that were not within ±2 SD of the mean were excluded from the analysis. The numbers of trials excluded were 16 for the total looking time data, 15 for the response latency data, and 16 for the HR differences data (see the Supplementary Electronic Information). The analyses were performed with SPSS (version 22; IBM Corporation, Armonk, NY, USA). The data of this study are provided in the Supplementary Data File.

This study was approved by the Institutional Animal Care and Use Committee of the University of Tokyo (Approval Number: 27–10), and the methods were performed according to their guidelines and regulations. The owners or caretakers of the horses gave consent prior to their participation. The participant horses were not deprived of food, and they remained in a familiar environment.

### Data accessibility

The data supporting this article are included in the Supplementary Electronic Information.

## Results

**Figure 2:**
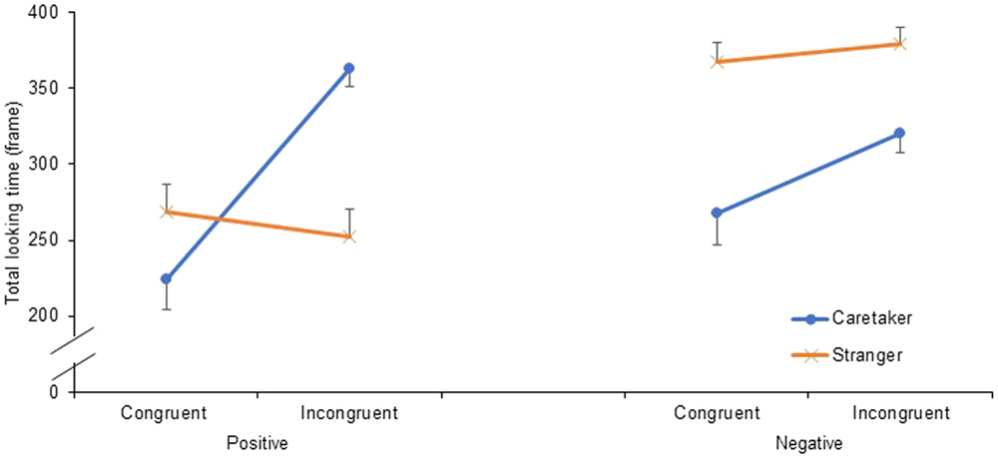
Mean ± standard error (SE) of the mean of the total looking time after presentation of the auditory stimulus.

Figure 2 shows the results for total looking time. The model included the fixed effects of emotional congruency, familiarity, emotional value of the visual stimulus, and their interactions and the random effects of participant identity, experimental place, and E2 [Akaike information criteria (AIC) = 1,649.093, Table 1]. The interaction between emotional congruency and familiarity was significant [*F*(1, 128) = 9.870, *p* = 0.002]. The horses looked toward the speaker for a significantly longer period in the incongruent condition compared with the congruent condition in the caretaker context [*t*(128) = 3.878, *p* < 0.001] and in the stranger context compared with the caretaker context in the congruent condition [*t*(128) = 1.990, *p* = 0.049]. However, the differences between the congruent and incongruent conditions in the stranger context [*t*(128) = 0.301, *p* = 0.764] and between the caretaker and stranger contexts in the incongruent condition [*t*(128) = 1.166, *p* = 0.246] were not significant in the multiple comparison tests conducted using the sequential Sidak’s method. Moreover, the interaction between familiarity and the emotional value of the visual stimulus was significant [*F*(1, 128) = 8.423, *p* = 0.004]. The horses looked toward the speaker for a significantly longer period in the stranger context compared with the caretaker context in the negative situation [*t*(128) = 3.359, *p* = 0.001] and in the negative situation compared with the positive situation in the stranger context [*t*(128) = 4.600, *p* < 0.001]. However, the differences between the caretaker context and stranger context in the positive situation [*t*(128) = 1.110, *p* = 0.269] and between the positive condition and negative condition in the caretaker condition were not significant [*t*(128) = 0.070, *p* = 0.945] in the multi*p*le comparison tests conducted using the sequential Sidak’s method. The interactions between emotional congruency and the emotional value of the visual stimulus [*F*(1, 128) = 0.414, *p* = 0.521] and among emotional congruency, familiarity, and the emotional value of the visual stimulus [*F*(1, 128) = 3.030. *p* = 0.084] were not significant.

**Table 1:** Linear mixed model results for total looking time.

Variables	Variance	*β*	SE	*t*	*p*	95% CI
Fixed factors						
Intercept		265.638	39.852	6.666	<0.001	186.784–344.493
Emotional congruency		27.958	44.762	0.625	0.533	−60.612–116.527
Familiarity		124.985	38.474	3.249	0.001	48.858–201.112
Emotional value		139.951	36.657	3.709	<0.001	63.419–208.483
Emotional congruency × Familiarity		−127.850	47.649	−2.683	0.009	−222.959– −32.742
Emotional congruency × Emotional value		−41.254	48.792	−0.846	0.399	−137.797–55.289
Familiarity × Emotional value		−182.943	47.831	−3.825	<0.001	−277.585–−88.301
Emotional congruency × Familiarity × Emotional value		131.093	75.306	1.741	0.084	−17.913–80.100
Random factors						
Horse identity	1725.267					
Experimental place	0					
E2	1869.019					

Abbreviations: SE, standard error of the mean; CI, confidence interval.

**Figure 3:**
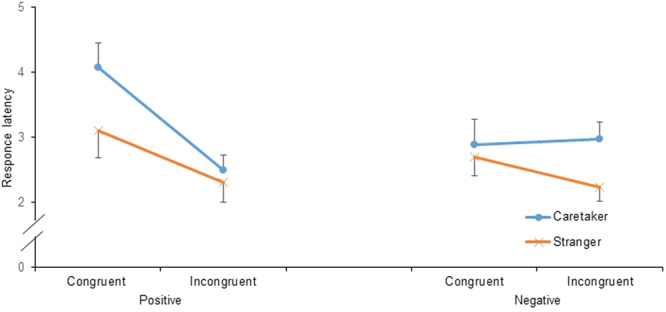
Mean ± SE response latency after presentation of the auditory stimulus. The raw data were log-transformed.

Figure 3 shows the results for response latency. The model included the fixed effects of emotional congruency, familiarity, the emotional value of the visual stimulus, and their interactions and the random effects of participant identity, experimental place, and E2 (AIC = 449.384, Table 2). As predicted, the main effect of emotional congruency was significant [*F*(1, 121) = 9.329, *p* = 0.003]. The horses responded significantly faster in the incongruent condition than in the congruent conditions. The main effect of familiarity was also significant [*F*(1, 121) = 6.310, *p* = 0.013]. The horses responded significantly faster in the stranger context than in the caretaker context. The main effects of the emotional value of the visual stimulus [*F*(1, 121) = 3.315, *p* = 0.071] and the interactions between emotional congruency and familiarity [*F*(1, 121) = 0.029, *p* = 0.866], between emotional congruency and the emotional value of the visual stimulus [*F*(1, 121) = 3.599, *p* = 0.060], between familiarity and the emotional value of the visual stimulus [*F*(1, 121) = 0.442, *p* = 0.507], and among emotional congruency, familiarity, and the emotional value of the visual stimulus [*F*(1, 121) = 1.290, *p* = 0.258] were not significant.

**Table 2:** Linear mixed model results for response latency.

Variables	Variance	*β*	SE	*t*	*p*	95% CI
Fixed factors						
Intercept		2.301	0.314	7.319	<0.001	1.679–2.923
Emotional congruency		0.792	0.520	1.521	0.131	−0.238–1.821
Familiarity		0.411	0.356	1.153	0.251	−0.294–1.117
Emotional value		−0.086	0.347	−0.247	0.805	−0.772–0.600
Emotional congruency × Familiarity		0.567	0.675	0.840	0.403	−0.770–1.904
Emotional congruency × Emotional value		−0.330	0.616	−0.536	0.593	−1.549–0.889
Familiarity × Emotional value		0.204	0.440	0.463	0.644	−0.667–1.074
Emotional congruency × Familiarity × Emotional value		−0.985	0.867	−1.136	0.258	−2.703–0.732
Random factors						
Horse identity	0					
Experimental place	0.018					
E2	0					

Abbreviations: SE, standard error of the mean; CI, confidence interval.

**Figure 4:**
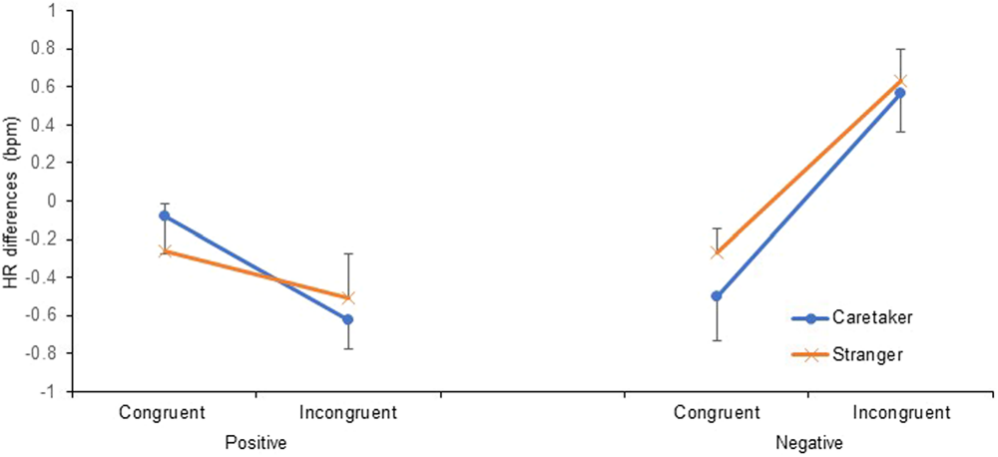
Mean ± SE difference in heart rate (HR) between immediately before and 15 s after presentation of the auditory stimulus.

Figure 4 shows the results for the difference in HR from immediately before to 15 s after presentation of the auditory stimulus. The model included the fixed effects of emotional congruency, familiarity, the emotional value of the visual stimulus, and their interactions and the random effects of participant ID, experimental place, and E2 (AIC = 480.923, Table 3). The interaction between emotional congruency and the emotional value of the visual stimulus was significant [*F*(1, 120) = 6.919, *p* = 0.010]. HR increased significantly more in the incongruent condition than in the congruent condition in the negative situation [*t*(120) = 2.900, *p* = 0.004] and in the negative situation than in the positive situation in the incongruent condition [*t*(120) = 3.310, *p* = 0.001]. However, the differences between the congruent and incongruent conditions in the positive situation [*t*(120) = 0.965, *p* = 0.337] and between the positive and negative situation in the congruent condition [*t*(120) = 0.564, *p* = 0.574] were not significant in the multiple comparison tests conducted using the sequential Sidak’s method. The main effects of familiarity [*F*(1, 120) = 0.022, *p* = 0.882] and the interactions between emotional congruency and familiarity [*F*(1, 120) = 0.019, *p* = 0.891], between familiarity and the emotional value of the visual stimulus [*F*(1, 120) = 0.028, *p* = 0.867], and among emotional congruency, familiarity, and the emotional value of the visual stimulus [*F*(1, 120) = 0.164, *p* = 0.686] were not significant.

**Table 3:** Linear mixed model results for the differences in heart rate (HR).

Variables	Variance	*β*	SE	*t*	*p*	95% CI
Fixed factors						
Intercept		−0.497	0.433	−1.146	0.254	−1.354–0.361
Emotional congruency		0.237	0.641	0.370	0.712	−1.032–1.507
Familiarity		−0.137	0.506	−0.270	0.787	−1.139–0.866
Emotional value		1.092	0.515	2.122	0.036	0.073–2.111
Emotional congruency × Familiarity		−0.648	1.096	−0.591	0.557	−2.837–1.542
Emotional congruency × Emotional value		−1.161	0.747	−1.555	0.123	−2.641–0.318
Familiarity × Emotional value		0.124	0.697	0.178	0.859	−1.257–1.504
Emotional congruency × Familiarity × Emotional value		−0.423	1.044	−0.405	0.686	−2.490–1.644
Random factors						
Horse identity	0.303					
Experimental place	0					
E2	0					

Abbreviations: SE, standard error of the mean; CI, confidence interval.

## Discussion

In the present study, our results for total looking time showed an interaction between emotional congruency and familiarity: the horses looked at the speaker significantly longer after listening to the auditory stimulus in the incongruent condition compared with the congruent condition only in the caretaker context. These results suggested that an expectancy violation occurred in the caretaker context, which supported our hypotheses for both the congruency and familiarity of the total looking time. In addition, we found that horses looked at the speaker significantly faster after listening to the auditory stimulus in the incongruent condition compared with the congruent condition in both the caretaker and stranger contexts, which partly supported our hypotheses. These results suggested that an expectancy violation occurred when the auditory stimulus had a different emotional value than the visual one because the horses were able to identify human emotions from the facial expressions^16^. Therefore, our results suggested that horses associate the emotional value of human facial expressions with the emotional value of human voices. These interpretations are plausible because horses cross-modally recognize familiar conspecifics and humans^21–23^, and the cross-modal perceptions of horses are thought to be flexible as our results suggested that they were able to do this even with strangers.

However, emotional congruency and familiarity did not have a significant interaction in the response latency results, though the effect of familiarity was significant. The horses responded to the human voices faster in the stranger context than in the caretaker context. These results did not support our hypothesis about familiarity bias. The participant horses should have been able to distinguish their caretaker from the stranger because horses can discriminate identical twins^28^ and familiar people from strangers, to which they pay more attention^29^. These findings suggested that the horses paid more attention to the stranger stimuli over the effects of the expectancy violation after distinguishing their own caretaker from the stranger.

For the total looking time results, familiarity and the emotional value of the visual stimulus had a significant interaction. In the stranger context, the horses looked at the speaker longer in the negative situation than in the positive situation. In the negative situation, the horses looked at the speaker longer in the stranger context than in the caretaker context. In the caretaker contexts, the horses were able to recognize his/her emotion by his/her voice even though his/her face disappeared because horses were able to match the caretaker’s voice with his/her face^22,23^. Therefore, expectancy violation occurred by the difference of the emotional value between the visual stimuli and auditory stimuli in the caretaker condition. On the other hand, in the stranger contexts, horses should not be able to initially associate a stranger’s face with his/her voice and the horses should not understand that the stimulus face and the stimulus voice of the stranger are from the same person. That is, horses were able to recognize that the stranger paid attention to themselves while his/her face stimulus continued to be presented. However, the horses would not recognize that the subsequent stranger’s voice stimulus was directed at themselves because the visual stimulus disappeared already and they were not be able to recognize that the auditory stimulus and the visual one were from the same person when they heard the auditory stimulus. Therefore, expectancy violation might not occur regardless of the visual stimulus’s emotion in the stranger context. However, the horses might respond sensitively and attend to the subsequent auditory stimulus in the negative condition, because negative emotion functions as threat and increases the horses’ tension even in the stranger context^16^.

For the physiological indexes, the difference in HR between immediately before and 15 s after presentation of the auditory stimulus and the interaction between emotional congruency and the emotional value of the visual stimulus were significant. HR increased more in the incongruent condition than in the congruent condition after the horses looked at the negative human facial expression, which suggested that an expectancy violation occurred. Because HR indicates degree of arousal^30,31^, this result suggested that the horses’ arousal increased^31^ when they heard a positive voice after looking at a negative facial expression, and they probably became nervous^16^, which suggested an expectancy violation had occurred. However, HR did not increase after the horses looked at a positive human facial expression in the positive situation, even though they were presented a negative voice, because they were probably relaxed after looking at the positive face^16^, and their arousal decreased^30^. Therefore, these results partially suggest that expectancy violations affect HR. However, the average HR from the start to the end of each trial decreased gradually from the trial start to the start of the auditory stimuli in this study, which indicated that the horses needed more habituation time with the experimental set-ups. Additionally, the experimenter who was standing beside the participant horse (E2) watched the HR receiver attached to the halter before the start of the trial in this study, which might have increased the horse’s tension. These explanations are plausible because horses are sensitive to human attentional states^32,33^ and human tension affects the HR of horses^34^. Therefore, their HRs might have started to decrease after the experimenter, who might have been nervous, stopped paying attention to the horses, which was the same time as the start of the trial. Nevertheless, because HR reflects degree of arousal, an increase in HR should indicate an increase in arousal resulting from an expectancy violation. Future experimental setups should be designed so that the horses are not affected by humans and their HR scores can be used as their expectancy violation index.

The results of a previous study suggested that dogs integrate visual and auditory emotional information^15^, which was consistent with our results. These findings imply that companion animals that have lived with humans for a long time have developed the ability to recognize human emotional states. However, comparing these results should be done cautiously because different methods were used in the two studies. That is, in the previous study, the preferential looking paradigm was used and the familiarity of the stimuli was not considered, while, in the present study, the expectancy violation method was used and we took the familiarity of the stimuli into account. Therefore, we should first test dogs and horses with a similar method (e.g., the expectancy violation method or the preferential looking paradigm) in order to compare the results directly and fairly. Nevertheless, few studies have examined cross-modal emotional perception in companion animals. Additional comparative studies of the other domesticated and the related non-domesticated animals are necessary before concluding about the evolution of cross-modal emotional perception in companion animals.

In this study, we reported the first evidence of the cross-modal perception of human emotion involving visual and auditory signals in horses, and our results suggested that the ability was generalized, even toward unfamiliar people. In addition, these results indicate that interactions involving emotional information, such as facial or voice expression, have played important roles in the social signals of horses throughout the history of their cooperative relationship with humans. Future studies should examine whether this cross-modal perception of emotion in horses is innate or learned to understand the effects of genetics and environment on the development of this ability^35^.

## Electronic supplementary material

Supplemental materials and methods

Dataset
