# Supplementary material for: Cross-modal perception of human emotion in domestic horses (Equus caballus)
Source: Sci Rep. 2018 Jun 21;8:8660. doi: 10.1038/s41598-018-26892-6 (PMC6013457; doi:10.1038/s41598-018-26892-6)
Supplement: Supplementary file 1 — Supplemental materials and methods [file 41598_2018_26892_MOESM1_ESM.pdf]

Supplemental materials and methods

Cross-modal perception of human emotion in domestic horses (*Equus caballus*)

Kosuke Nakamura[1], Ayaka Takimoto[2, 3], Toshikazu Hasegawa[1]

[1] Department of Cognitive and Behavioral Sciences, Graduate School of Arts and Sciences, The University of Tokyo.
[2] Department of Behavioral Sceiences, Graduate School of Letters, Hokkaido University
[3] Center for Experimental Research in Social Sciences, Hokkaido University

1. Additional methods

a) Stimuli recording and validation

Visual stimuli were taken using a digital camera (A3300, SONY). Pictures were edited with Adobe Photoshop Element 12 to be the height of 1500 pixels after trimmed so that the vertical width was from the head top to the chin and the horizontal width was the width of the left and right ears and background was filled with white. Visual stimuli (about A3 size; 42 × 29.7 cm) were presented with the projector (V-1080 PLUSVision) to the screen (135 × 180 cm) (referring to Smith et al[1]). A happy face as a positive stimulus and an anger face as a negative stimulus were taken from the front. Facial expressions were validated using Facial Action Coding System (FACS) descriptives[2]. Stimulus people were instructed to pull up their lip corner and raise cheek when taking the happy face photograph and to frown and glare at the camera when taking the angry face.

Auditory stimuli were recorded with the digital voice recorder (ICR-PS501RM, SANYO). The Auditory cue was the nickname of the participant horse. A gentle call as a positive stimulus and a scolding tone of voice as a negative stimulus were recorded. Stimulus people were instructed to imagine praising horses when recording positive stimuli and scolding horses playing a trick when recording negative stimuli. Auditory stimuli were edited with SoundEngine ver.5.21 to play three times putting in 1 second blank between each call. Stimuli were presented from the speaker (SoundLink Mobile Ⅱ-Leather BOSE) and were 67 ± 2.1 dB measured from the subjects' position. The average length of the stimulus voices was 5.4 ± 0.67 sec. The length of them was different depending upon the length of the participant nickname from 4 to 8 sec.

Visual and auditory stimuli were rated by five people who were independent to the experiment. All stimuli were rated appropriately.

b) Equipment

The experiment was conducted in a vacant stable in the stables of the equestrian team of the University of Tokyo and the equestrian team of the Tokyo University of Agriculture and Technology. The size of the experimental place was 3.2 × 2.4 × 3.7m in the University of the Tokyo and 3.0 × 3.0 × 4.5m in Tokyo University of Agriculture and Technology. The screen was placed outside the experimental place at a distance of 1.3m and the speaker was set in front of it.

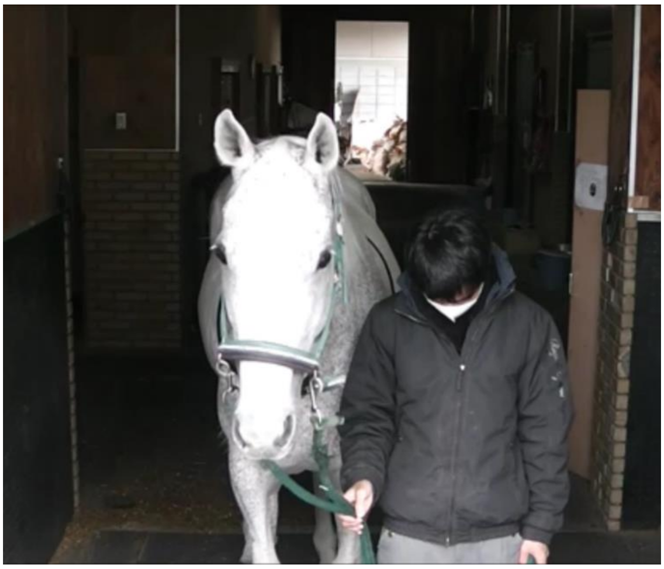

Figure S1. Photograph of a horse participating in the experiment, taken from the front video camera.

c) Behavioral coding scheme

Definitions of behaviorally coded variables are based on Lampe and Andre[3].

Table S1. Definitions of behaviorally coded variables.

Behavior	Coding scheme definition
Looking	When a horse faced the nostrils ≤45° to the right or to the left of the speaker and had at least one moment (of ≥120 ms) of gazing fixedly. The “beginning” or “end” of a look was defined when the horse’s head started to move into or out of the ≤45° zone, respectively. The 45° angle was reached when (a) the horse’s eyeball facing the loud speaker disappeared with only the curve of the eye socket remaining visible and (b) the nostril of that same side was out of sight. In some trials, subjects were already holding their heads at a ≤45° angle to the speaker when the auditory cue started to play. If in these trials a horse kept looking in the direction of the speaker after the onset of the voice tape and either started (a) to narrow the angle to the speaker and/or (b) to blink, this was counted as the beginning of a ‘‘look.’’[3]
Behavioral index	
Response latency	The latency from the start of the auditory stimulus to the looking.
Total looking time	Looking time between the start of the auditory stimulus and the end of the trial.

d) Analysis

A linear mixed model was used to examine the effects of emotional congruency, familiarity, and visual stimulus emotional value. Data that were not within ± 2 SD of the mean were excluded from the analysis. The numbers of trials excluded were 16 for the total looking time data (2 for congruent × caretaker × positive treatment, 1 for congruent × caretaker × negative treatment, 2 for incongruent × caretaker × positive treatment, 3 for incongruent × caretaker × negative treatment, 3 for congruent × stranger × positive treatment, 3 for congruent × stranger × negative treatment and 2 for incongruent × stranger × negative treatment), 15 for the response latency data (1 for congruent × caretaker × positive treatment, 1 for congruent × caretaker × negative treatment, 2 for incongruent × caretaker × positive treatment, 2 for incongruent × caretaker × negative treatment, 3 for congruent × stranger × positive treatment, 2 for congruent × stranger × negative treatment, 1 for incongruent × stranger × positive treatment and 3 for incongruent × stranger × negative treatment), and 16 for the HR differences data (2 for congruent × caretaker × positive treatment, 2 for congruent × caretaker × negative treatment, 3 for incongruent × caretaker × positive treatment, 2 for incongruent × caretaker × negative treatment, 3 for congruent × stranger × positive treatment, 1 for congruent × stranger × negative treatment, 1 for incongruent × stranger × positive treatment and 2 for incongruent × stranger × negative treatment). The analyses were performed with SPSS (version 22; IBM Corporation, Armonk, NY, USA).

Table S2. The number of the excluded data per treatment

Emotional value	Positive				Negative			
Familiarity	Caretaker		Stranger		Caretaker		Stranger	
Emotional congruency	congruent	incongruent	congruent	incongruent	congruent	incongruent	congruent	incongruent
Total looking time	2	1	2	3	3	3	0	2
Response latency	1	1	2	2	3	2	1	3
HR differences	2	2	3	2	3	1	1	2

e) Additional HR information

HR was analyzed with the software "POLAR FLOW" by 1 sec. The HR difference between immediately before and after 15 secs from presenting auditory stimulus was analyzed.

2. Additional results

TableS3 shows the data of the independent variance. TableS4~6 shows the fixed effect of the independent variance.

Table S3. Average value (SE) of the independent variance

Emotional value	Positive				Negative			
Familiarity	Caretaker		Stranger		Caretaker		Stranger	
Emotional congruency	congruent	incongruent	congruent	incongruent	congruent	incongruent	congruent	incongruent
Total looking time	225 (40.7)	363 (25.1)	269 (37.0)	252 (37.3)	268. (42.3)	320 (25.8)	367 (25.6)	380 (21.4)
Response latency	4.07 (0.383)	2.49 (0.226)	3.10 (0.424)	2.30 (0.300)	2.89 (0.397)	2.96 (0.272)	2.69 (0.288)	2.23 (0.207)
HR differences	-0.084 (0.379)	-0.622 (0.313)	-0.259 (0.484)	-0.505 (0.454)	-0.502 (0.469)	0.563 (0.403)	-0.271 (0.248)	0.631 (0.327)

Table S4 Fixed effect of total looking time

	F value	P
Emotional congruency	*F* (1, 128) = 7.653	.003
Familiarity	*F* (1, 128) = 1.880	.173
Emotional value	*F* (1, 128) = 9.229	.003
Emotional congruency × Familiarity	*F* (1, 128) = 9.870	.002
Emotional congruency × Emotional value	*F* (1, 128) = 0.414	.521
Familiarity × Emotional value	*F* (1, 128) = 8.423	.004
Emotional congruency × Familiarity × Emotional value	*F* (1, 128) = 3.030	.084

Table S5. Fixed effect of response latency

	F value	P
Emotional congruency	*F* (1, 121) = 9.329	.003
Familiarity	*F* (1, 121) = 6.310	.013
Emotional value	*F* (1, 121) = 3.315	.071
Emotional congruency × Familiarity	*F* (1, 121) = 0.029	.866
Emotional congruency × Emotional value	*F* (1, 121) = 3.599	.060
Familiarity × Emotional value	*F* (1, 121) = 0.442	.507
Emotional congruency × Familiarity × Emotional value	*F* (1, 121) = 1.290	.258

TableS6. Fixed effect of HR differences

	F value	P
Emotional congruency	F (1, 120) = 1.393	.240
Familiarity	F (1, 120) = 0.022	.882
Emotional value	F (1, 120) = 3.219	.075
Emotional congruency × Familiarity	F (1, 120) = 0.019	.891
Emotional congruency × Emotional value	F (1, 120) = 6.919	.010
Familiarity × Emotional value	F (1, 120) = 0.028	.867
Emotional congruency × Familiarity × Emotional value	F (1, 120) = 0.164	.686

Supplementary references

1. Smith, A. V., Proops, L., Grounds, K., Wathan, J., & McComb, K. Functionally relevant responses to human facial expressions of emotion in the domestic horse (*Equus caballus*). *Biol Let*, **12(2)**, 20150907. http://doi.org/10.1098/rsbl.2015.0907 (2016)
2. Ekman P, Friesen WV. *Facial action coding system: A technique for the measurement of facial movement*. Palo Alto: Consulting Psychologists Press. (1978)
3. Lampe, J. F., & Andre, J. Cross-modal recognition of human individuals in domestic horses (*Equus caballus*). *Anim Cogn*, **15(4)**, 625-626. http://doi.org/10.1007/s10071-012-0490-1 (2012)
